# Silicon-Mediated Alleviation of Aluminum Toxicity by Modulation of Al/Si Uptake and Antioxidant Performance in Ryegrass Plants

**DOI:** 10.3389/fpls.2017.00642

**Published:** 2017-04-25

**Authors:** Sofía Pontigo, Karina Godoy, Héctor Jiménez, Ana Gutiérrez-Moraga, María de la Luz Mora, Paula Cartes

**Affiliations:** ^1^Programa de Doctorado en Ciencias de Recursos Naturales, Universidad de La FronteraTemuco, Chile; ^2^Center of Plant-Soil Interaction and Natural Resources Biotechnology, Scientific and Technological Bioresource Nucleus (BIOREN-UFRO), Universidad de La FronteraTemuco, Chile; ^3^Departamento de Producción Agropecuaria, Facultad de Ciencias Agropecuarias y Forestales, Universidad de La FronteraTemuco, Chile; ^4^Departamento de Ciencias Químicas y Recursos Naturales, Facultad de Ingeniería y Ciencias, Universidad de La FronteraTemuco, Chile

**Keywords:** silicon, aluminum, Si transporter genes, phenols, antioxidant enzymes, SOD isoforms genes

## Abstract

Silicon (Si) has been well documented to alleviate aluminum (Al) toxicity in vascular plants. However, the mechanisms underlying these responses remain poorly understood. Here, we assessed the effect of Si on the modulation of Si/Al uptake and the antioxidant performance of ryegrass plants hydroponically cultivated with Al (0 and 0.2 mM) in combination with Si (0, 0.5, and 2.0 mM). Exposure to Al significantly increased Al concentration, mainly in the roots, with a consequent reduction in root growth. However, Si applied to the culture media steadily diminished the Al concentration in ryegrass, which was accompanied by an enhancement in root dry matter production. A reduced concentration of Si in plant tissues was also observed when plants were simultaneously supplied with Al and Si. Interestingly, Si transporter genes (*Lsi1* and *Lsi2*) were down-regulated in roots after Si or Al was applied alone; however, both *Lsi1* and *Lsi2* were up-regulated as a consequence of Si application to Al-treated plants, denoting that there is an increase in Si requirement in order to cope with Al stress in ryegrass. Whereas Al addition triggered lipid peroxidation, Si contributed to an attenuation of Al-induced oxidative stress by increasing phenols concentration and modulating the activities of superoxide dismutase (SOD), catalase, peroxidase, and ascorbate peroxidase antioxidant enzymes. Differential changes in gene expression of SOD isoforms (*Mn-SOD, Cu/Zn-SOD*, and *Fe-SOD*) and the profile of peroxide (H_2_O_2_) generation were also induced by Si in Al-stressed plants. This, to the best of our knowledge, is the first study to present biochemical and molecular evidence supporting the effect of Si on the alleviation of Al toxicity in ryegrass plants.

## Introduction

Aluminum (Al) toxicity represents one of the main yield-limiting factors for crops in acid soils (von Uexküll and Mutert, 1995). Under acidic conditions, large and toxic amounts of Al^3+^ become available to plants, thereby affecting a wide range of physical, cellular, and molecular processes, with a consequent reduction in plant growth (Kochian et al., 2005; Mora et al., 2006; Cartes et al., 2010, 2012; Ryan and Delhaize, 2010; Singh et al., 2017). Alterations in the structure and/or functions of cell wall components (Horst et al., 2010), plasma membrane properties (Yamamoto et al., 2001), nutrient homeostasis (Delhaize and Ryan, 1995; Gupta et al., 2013; Singh et al., 2017), and signal transduction pathways (Matsumoto, 2000; Ma et al., 2002; Sivaguru et al., 2003; Goodwin and Sutter, 2009) can be induced as a consequence of Al binding to numerous cell sites. In most plant species, reactive oxygen species (ROS) production can also be induced by Al toxicity (Kochian et al., 2005), leading to oxidative damage of biomolecules and biological membranes (Yamamoto et al., 2001, 2002, 2003; Singh et al., 2017).

To cope with the deleterious effects of Al, plant species have developed diverse mechanisms, which are generally associated with Al exclusion (also referred to as avoidance or resistance) and/or internal tolerance mechanisms (e.g., Barcelo and Poschenrieder, 2002; Kochian et al., 2005; Poschenrieder et al., 2008). Briefly, exclusion mechanisms involve the root exudation of organic acid anions and/or phenolic compounds, which bind Al^3+^ and limit its uptake into the cytosol. Tolerance mechanisms comprise internal detoxification by forming Al complexes with organic substances in the cytosol, compartmentalization in the vacuole, and enhanced scavenging of ROS (e.g., Barcelo and Poschenrieder, 2002; Kochian et al., 2005; Poschenrieder et al., 2008). Molecular approaches have revealed that Al resistance in several plant species is regulated by genes encoding membrane transporter proteins involved in the efflux of organic acid anions, including members of the ALMT (aluminum-activated malate transporters) and MATE (multidrug and toxic compound extrusion) families (Sasaki et al., 2004; Furukawa et al., 2007; Ryan et al., 2011). In addition, a bacterial-type ATP binding cassette (ABC) transporter (Huang et al., 2009) and antioxidant defense genes (e.g., Milla et al., 2002; Goodwin and Sutter, 2009; Du et al., 2010; Panda and Matsumoto, 2010) have also been implicated in Al tolerance in plants.

Over the last decades, silicon (Si) has become a focus of increasing interest in plant science, since it is considered as a beneficial element for plant growth, particularly under conditions of biotic and abiotic stress (Ma, 2004; Liang et al., 2007; Guntzer et al., 2012; Ma and Yamaji, 2015). To date, several pieces of evidence have indicated that most of the beneficial effects of Si depend on the differential ability of plants to take up Si. Recently, it has been reported that Si accumulation is ascribed to an efficient uptake system mediated by both channel-type and efflux transporters, which perform coordinated functions for effective Si transport from soil to roots and its subsequent distribution within the plants (e.g., Ma et al., 2006, 2007; Yamaji et al., 2008, 2012; Chiba et al., 2009; Mitani et al., 2009a,b, 2011a,b; Yamaji and Ma, 2009; Grégoire et al., 2012; Montpetit et al., 2012; Deshmukh et al., 2013; Ma and Yamaji, 2015). Overall, these transporters appear to be keys features that enable plants to gain an advantage from Si uptake. Nevertheless, the regulation of Si transporters under stress conditions remains poorly understood.

The significant role of Si in the toxicity associated with metals, including manganese (Mn), iron (Fe), cadmium (Cd), arsenic (As), chromium (Cr), copper (Cu), lead (Pb), zinc (Zn), and Al, has been widely reported (Li et al., 2012; Vaculík et al., 2012; Adrees et al., 2015; Liang et al., 2015; Pontigo et al., 2015; Tripathi et al., 2015, 2016). On the basis of the current evidence, Si can regulate plant resistance and/or tolerance to metal toxicity by either external (*ex planta*) or internal (*in planta*) mechanisms (Cocker et al., 1998a; Adrees et al., 2015; Liang et al., 2015; Pontigo et al., 2015; Tripathi et al., 2016). In this regard, it has been proposed that the alleviation of Al stress by Si in plants can mainly be explained by the following events: (i) Si-induced increase in solution pH (Li et al., 1996; Cocker et al., 1998a), (ii) formation of Al-Si complexes in the growth media (Barcelo et al., 1993; Baylis et al., 1994; Ma et al., 1997; Cocker et al., 1998a) or/and within the plant (Corrales et al., 1997; Cocker et al., 1998b; Britez et al., 2002; Zsoldos et al., 2003; Wang et al., 2004; Prabagar et al., 2011), (iii) exudation of organic acid anions and phenolic compounds (Barcelo et al., 1993; Cocker et al., 1998b; Kidd et al., 2001), and (iv) increase in the chlorophyll and carotenoid contents of leaves (Singh et al., 2011). Activation of the plant antioxidant system has also been reported in response to Si supply under Al stress (Shahnaz et al., 2011; Shen et al., 2014; Tripathi et al., 2016). However, to our knowledge, there is a dearth of reports regarding the molecular aspects of the effect of Si on the genes involved in antioxidant defense.

Perennial ryegrass (*Lolium perenne* L.) is a temperate pasture species supporting forage-based intensive dairy and beef production systems in many parts of the world. Due to elevated yields and high nutritional value, ryegrass has become one of the most commonly cultivated species in the permanent pastures of Southern Chile. Nevertheless, large areas of these pastures are sown on acidic soils, which exhibit elevated availability of toxic Al^+3^, thereby limiting their yield and quality (Mora et al., 2006). Furthermore, our previous studies have demonstrated that toxic levels of Al induced oxidative damage and activated antioxidant enzymes in ryegrass roots, including peroxidase (POD), ascorbate peroxidase (APX), and superoxide dismutase (SOD) (Cartes et al., 2010, 2012). In an attempt to identify new alternatives to alleviate the deleterious effects produced by Al on ryegrass, we aimed in this study to investigate the effect of Si on the modulation of Si/Al uptake and the antioxidant performance of ryegrass plants subjected to Al toxicity.

## Materials and Methods

### Plant Material and Growth Conditions

Seeds of ryegrass (*L. perenne* L. cultivar Nui) were soaked with 2% v/v sodium hypochlorite for 10 min, washed repeatedly with distilled water, and then germinated on moist filter paper in a growth chamber at 21°C. After 10 days, seedlings were transferred to 12-L plastic pots containing a continuously aerated basal nutrient solution described by Taylor and Foy (1985). After 10 days in nutrient solution, ryegrass plants were treated with Al and Si. Aluminum (as AlCl_3_, Merck reagent) was added to the solution at doses of 0 and 0.2 mM. The activity of free Al^3+^ in the nutrient solution, calculated by Geochem-EZ (Shaff et al., 2010), corresponded to 85 μM. Aluminum doses were added in combination with 0, 0.5, and 2 mM Si (as Na_2_SiO_3,_ Merck reagent) in a completely randomized factorial design with three replicates per treatment. During the growth period, the pH of the solution was adjusted daily to 4.5 using dilute HCl or NaOH, and the nutrient solution was changed every 7 days. Plants were cultured in a greenhouse under controlled growth conditions as follows: 25/20°C day/night temperature, a 16/8 h (light/dark) photoperiod, 350 μmol m^-2^ s^-1^ photosynthetic photon flux (PPF) and 70–80% relative humidity. Plants were harvested 10 days after the initiation of treatments, and shoot and root samples were stored at -20°C or -80°C for subsequent evaluation of biochemical and molecular parameters. In addition, subsamples of fresh material were dried at 65°C for 48 h in order to determinate the dry weight as well as Si and Al concentrations.

### Determination of the Mineral Concentration of Al and Si in Plant Tissues

Aluminum analysis was performed on dried roots and shoots. Plant samples were ashed at 500°C for 8 h and treated with 2 M HCl. After filtration of the resulting solution, the total amount of Al was quantified by flame atomic absorption spectrophotometry (FAAS) at 324.7 nm, as described by Sadzawka et al. (2007). Silicon concentration was assayed as described by Pavlovic et al. (2013) with modifications. Dry plant samples were digested with 5 mL concentrated HNO_3_ on a hot plate at 70°C for approximately 5 h. Samples were diluted with 10 mL of deionized water, followed by the addition of 1 mL HF (40%), and left overnight. The following day, 5 mL 2% (w/v) H_3_BO_3_ was added to eliminate excess HF and the volume of the solution was adjusted to 25 mL with deionized water. The Si concentration in the digested samples was determined by FAAS at 251.6 nm. For each chemical analysis, two reference samples were included in each analytical run.

### Biochemical Analyses

#### Lipid Peroxidation Assay

Lipid peroxidation was analyzed using the thiobarbituric acid reactive substances (TBARS) assay, according to the modified method of Du and Bramlage (1992). The absorbance of the samples was measured at 532, 600, and 440 nm in order to correct for interference generated by TBARS-sugar complexes.

#### Determination of Total Phenols

Total soluble phenols were spectrophotometrically assayed at 765 nm using Folin-Ciocalteu reagent according to the method described by Slinkard and Singleton (1977) with minor modifications (Ribera et al., 2013). Total phenol concentration was calculated using chlorogenic acid as a phenolic compound standard.

#### Antioxidant Enzyme Assays

SOD (EC. 1.15.1.1), catalase (CAT; EC. 1.11.1.6), peroxidase (POD; EC. 1.11.1.7), and APX (EC. 1.11.1.11) enzyme activities were evaluated from frozen samples stored at -80°C. Plant material was ground in liquid nitrogen and macerated in 50 mM potassium phosphate buffer (K_2_HPO_4_–KH_2_PO_4_; pH 7.0). The homogenate was centrifuged at 11,000 × *g* for 15 min at 4°C, and the supernatant was used for assay of enzyme activities. SOD, CAT, APX, and POD activities were calculated on a protein basis. The protein content in the extracts was measured spectrophotometrically using the method described by Bradford (1976), with bovine serum albumin (BSA) used as a standard.

Superoxide dismutase activity was analyzed by measuring inhibition of the photochemical reduction of nitroblue tetrazolium (NBT). The reaction mixture contained 400 μL of 0.1 M potassium phosphate buffer pH 7.0, 10 μL of 10 mM ethylenediaminetetraacetic acid (EDTA), 50 μL of 260 mM methionine, 80 μL of 4.2 mM NBT, 170 μL of 130 μM riboflavin, and 300 μL of enzyme extract. The reaction tubes were illuminated for 15 min and the absorbance of samples was measured at 560 nm. Non-illuminated and illuminated reactions without enzyme extract were used as controls. One SOD unit was defined as the amount of enzyme corresponding to 50% inhibition of NBT reduction (Donahue et al., 1997).

Catalase (CAT; EC. 1.11.1.6) activity was measured by monitoring the decomposition of hydrogen peroxide (H_2_O_2_) at 240 nm for 120 s. A 10-μL aliquot of enzyme extract was added to a reaction mixture containing 1 mL of extraction buffer and 3 μL of H_2_O_2_ (30% v/v). The enzyme activity was calculated using a molar extinction coefficient of 39.4 mM^-1^ cm^-1^ (Pinhero et al., 1997).

Peroxidase (POD; EC. 1.11.1.7) activity was determined by estimating the formation of tetraguaiacol at 470 nm during 1 min. A 15-μL volume of enzyme extract was added to a reaction mixture containing 1 mL of extraction buffer, 5 μL of H_2_O_2_ (30% v/v), and 5 μL of guaiacol. A molar extinction coefficient of 26.6 mM^-1^ cm^-1^ was used to calculate the enzymatic activity (Pinhero et al., 1997).

Ascorbate peroxidase (EC. 1.11.1.11) activity was assayed according to the method described by Nakano and Asada (1981), by measuring ascorbate decomposition at 290 nm for 1 min. The coarse extract (40 μL) was diluted in a reaction mixture containing 1 mL of extraction buffer, 5 μL of H_2_O_2_ (30% v/v), and 40 μL of 10 mM ascorbic acid. Enzyme activity was calculated using a molar extinction coefficient of 2.8 mM^-1^ cm^-1^.

### Gene Expression Analyses

Ryegrass tissues were subjected to RNA extraction using a NucleoSpin^®^ RNA Plant Kit (Macherey-Nagel GmbH and Co., KG, Düren, Germany). First-strand cDNA was synthesized from 1 μg of total RNA using an AffinityScript qPCR cDNA Synthesis Kit (Stratagene, Cedar Creek, TX, USA) following the manufacturer’s recommendations. Quantitative real-time polymerase chain (qRT-PCR) reactions were conducted in order to determinate the expression patterns of Si transporter genes (*Lsi1* and *Lsi2*) in roots, as well as those of three SOD isoform genes (*Cu/ZnSOD, Fe-SOD*, and *Mn-SOD*) in shoots and roots. All qRT-PCR reactions were performed using Brilliant II SYBR Green qPCR Master mix (Stratagene, Cedar Creek, TX, USA) in an ABI 7300 Real-Time PCR System (Applied Biosystems, Foster City, CA, USA). Cycling conditions were 95°C for 10 min, followed by 40 cycles at 95°C for 30 s, 60°C for 1 min, and 72°C for 30 s. The specific primers used in this study are shown in **Table 1**. The primer sets used for *LpLsi1* (GenBank accession number KY315994) and *LpLsi2* (GenBank accession number KY315995) were designed using the Primer3 (v. 0.4.0) and primer BLAST tools. Primers sequences for *LpCu/ZnSOD, LpFe-SOD*, and *LpMn-SOD* were obtained from Ribera et al. (2013). Housekeeping genes, *LpActin* or *LpeEF1A (m)*, were used as internal controls (Ribera et al., 2013). All the experiments were performed using three biological replicates, each with three technical replicates.

**Table 1:** List of primers sequences used for quantitative real-time polymerase chain reaction (qRT-PCR) analysis of Si transporters and SOD isoforms genes.

Gene name^∗^	Forward primer (5′- > 3′)	Reverse primer (5′- > 3′)
Lsi1	ACGCCCAGCATGTACTACAAC	TCATGAACACCAGCAGGAAC
Lsi2	CTCTGCATGTACTGGAAGGAC	GTTGAGAGGGTTGAGAGTGTG
Fe-SOD	GTTGCCAAGGGAAATCCTGAACCA	AACCCCAGCCGTTTATCTTCAAGC
Cu/Zn-SOD	GTGTTGCTCCCATCAATGTTGT	CCTGCCAAGATCATCAGCATC
Mn-SOD	AATACGAAAATGTGGCTGTGTG	AAAATCTGCATTGTGCATTACG
Actin	CCTTTTCCAGCCATCTTTCA	GAGGTCCTTCCTGATGTCCA
eEF1A (m)	GGCTGATTGTGCTGTGCTTA	CTCACTCCAAGGGTGAAAGC

*^∗^Gene name: *Lsi1*, Low Si transporter 1; *Lsi2*, Low Si transporter 2; *Fe-SOD*, iron superoxide dismutase; *Cu/Zn-SOD*, copper/zinc superoxide dismutase; *Mn-SOD*, manganese superoxide dismutase; *Actin*, Actin; *eEF1A(m)*, Eukaryotic elongation factor 1 alpha. *Actin* or *eEF1A(m)* were used as housekeeping genes.*

### Detection of H_2_O_2_ Production by Flow Cytometry

Suspensions of shoot protoplasts were obtained using the method described by Okuno and Furusawa (1977). The protoplasts were centrifuged at 2,500 × *g* for 5 min at 4°C and incubated with the fluorescent probe 2′,7′-dichlorodihydrofluorescein diacetate (H_2_DCFDA) to detect intracellular H_2_O_2_ using the method described by Maxwell et al. (1999) with modifications. H_2_O_2_ production was analyzed using flow cytometry (BD FACS Canto IISN: V96101286; Becton Dickinson, USA). All measurements were performed using an Ar ion laser excited at 488 nm and emitting at 530 nm. The images were processed through the BD FACSDivaTM, v 6.0 program. A positive control (intact protoplasts plus 100 μM H_2_O_2_) and negative control (suspension of intact protoplasts without H_2_O_2_) were used.

### Confocal Microscopy

A profile of H_2_O_2_ generation in protoplast extracts was also examined by Laser Scanning Confocal Microscopy (CLSM). H_2_DCFDA fluorescence emission was recorded at excitation/emission of 488/530 nm, and chlorophyll autofluorescence was measured at 633 nm laser excitation and emission of 750 nm. The images were processed using Image Processing software (software FV10-ASW v.0.2c; Arquimed).

### Statistical Analysis

Experimental data were analyzed using an analysis of variance (ANOVA) following normality and homoscedasticity tests. Differences among means were separated using the Tukey test at the 0.05 probability level. In addition, the relationship between two response variables was investigated by Pearson correlation.

## Results

### Concentrations of Al and Si in Plants and Dry Matter Production

Aluminum treatment mostly increased Al concentration in roots, whereas significantly lower amounts of Al accumulated in the shoots (**Table 2**). However, increasing Si doses gradually decreased shoot and root Al concentrations by up to 49 and 56%, respectively, in Al-treated plants (**Table 2**). Interestingly, a negative correlation between Si concentration and Al concentration was observed in shoots (*r* = 0.927, *p* ≤ 0.01) and roots (*r* = 0.935, *p* ≤ 0.01) of ryegrass grown with Al and Si (**Table 3**). In addition, the Si concentration of ryegrass tissues steadily increased with an increase in Si dose, but this increment was less noticeable when plants were simultaneously supplied with Al and Si (**Table 2**). Of the total amount of Si taken up by plants, over 80% accumulated in the shoots.

**Table 2:** Concentration of Al and Si, and dry matter production of ryegrass plants hydroponically cultivated under different Al and Si treatments.

Treatment (mM)	Al concentration (g kg^-1^ DW)		Si concentration (g kg^-1^ DW)		Dry weight (g)	
	Shoots	Roots	Shoots	Roots	Shoots	Roots
0 Al – 0 Si	0.02 ± 0.00cd	0.16 ± 0.02d	0.31 ± 0.09e	0.33 ± 0.03e	6.53 ± 0.29bc	1.37 ± 0.06ab
0 Al – 0.5 Si	0.01 ± 0.00d	0.15 ± 0.00d	5.85 ± 0.44c	6.42 ± 0.20c	7.04 ± 0.29abc	1.37 ± 0.10ab
0 Al – 2 Si	0.01 ± 0.00d	0.13 ± 0.01d	13.78 ± 0.26a	13.47 ± 0.09a	6.69 ± 0.22abc	1.39 ± 0.13a
0.2 Al – 0 Si	0.07 ± 0.00a	3.84 ± 0.24a	0.21 ± 0.03e	0.38 ± 0.10e	6.07 ± 0.42c	0.98 ± 0.06b
0.2 Al – 0.5 Si	0.04 ± 0.00b	2.68 ± 0.10b	4.40 ± 0.13d	4.30 ± 0.15d	7.95 ± 0.42ab	1.48 ± 0.08a
0.2 Al – 2 Si	0.03 ± 0.00bc	1.69 ± 0.11c	10.29 ± 0.19b	11.88 ± 0.20b	8.09 ± 0.32a	1.61 ± 0.06a

*Values are means ± standard error of three replicates. Different letters indicate statistically significant differences (*p* ≤ 0.05) among treatments.*

**Table 3:** Pearson’s correlation among plant growth, chemical and biochemical parameters of ryegrass hydroponically cultivated under different Al and Si treatments.

	Al	Si	Dry weight	TBARS	Total phenols	SOD	CAT	POD	APX
**Shoots**									
Al	1.00								
Si	-0.927**	1.00							
Dry weight	-0.849**	0.721*	1.00						
TBARS	0.946**	-0.947**	-0.757*	1.00					
Total phenols	-0.904**	0.859**	0.756*	-0.813**	1.00				
SOD	0.693*	-0.827**	-0.432	0.646	-0.721*	1.00			
CAT	-0.099	0.076	-0.118	-0.023	0.418	-0.110	1.00		
POD	0.863**	-0.776*	-0.781*	0.715*	-0.932**	0.666	-0.275	1.00	
APX	0.823**	-0.599	-0.745*	0.657	-0.744*	0.489	-0.073	0.836**	1.00
**Roots**									
Al	1.00								
Si	-0.935**	1.00							
Dry weight	-0.876**	0.823**	1.00						
TBARS	0.740*	-0.734*	-0.800**	1.00					
Total phenols	-0.825**	0.741*	0.706*	-0.523	1.00				
SOD	0.883**	-0.961**	-0.778*	0.787*	-0.731*	1.00			
CAT	-0.691*	0.838**	0.524	-0.399	0.738*	-0.795*	1.00		
POD	-0.796*	0.925**	0.666	-0.509	0.690*	-0.858**	0.956**	1.00	
APX	-0.925**	0.980**	0.806**	-0.666	0.800**	-0.930**	0.894**	0.962**	1.00

*Asterisks indicate significance as follows: ^∗∗^*p* ≤ 0.01,^∗^*p* ≤ 0.05.*

No changes in shoot growth were observed in plants treated with Al alone, whereas root dry matter production was reduced by approximately 28.5%. Silicon treatments did not affect ryegrass growth when Si was applied to plants cultivated without Al (**Table 2**). However, root yield was improved by at least 51% when Si was applied to Al-treated plants. Moreover, a positive correlation (*r* = 0.823, *p* ≤ 0.01) between Si concentration and dry weight was observed for the roots of Al-treated plants supplied with increasing concentrations of Si (**Table 3**).

### Analysis of Si Transporter Gene Expression in Response to Al Toxicity

**FIGURE 1:**
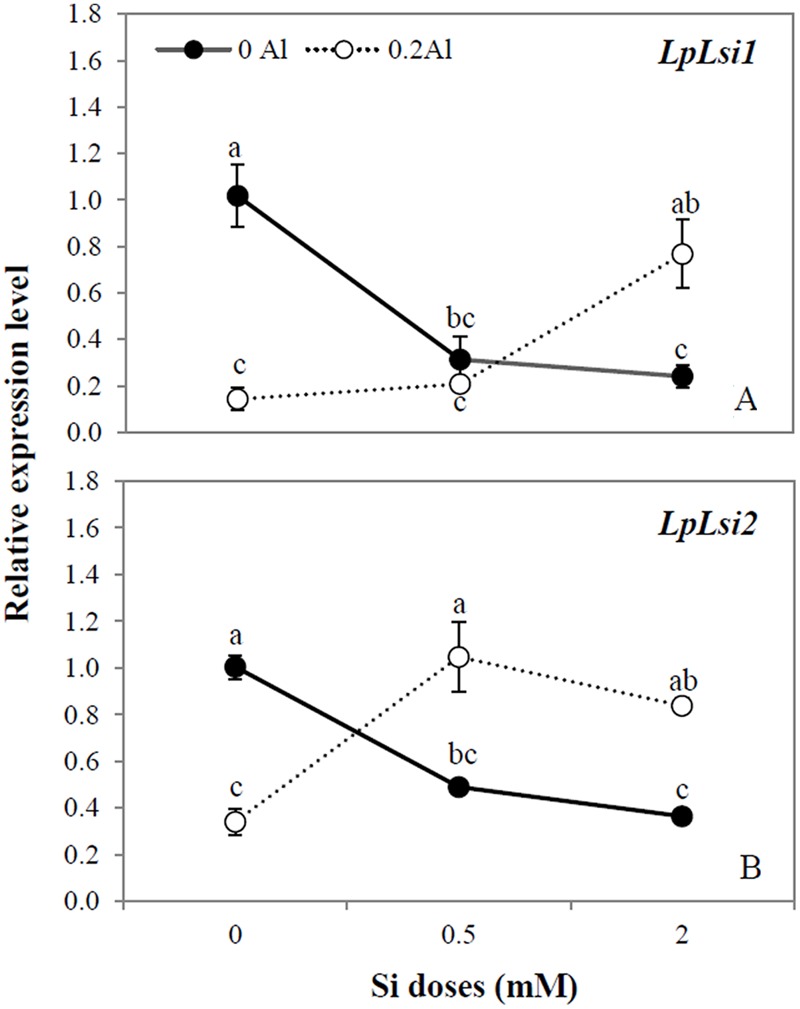
**Expression analysis of *LpLsi1* (A)** and *LpLsi2* **(B)** genes determined by qRT-PCR in roots of ryegrass hydroponically cultivated under Al and Si treatments. The expression levels were normalized in relation to *Actin* or *eEF1A(m)* gene expression. Data are means of three replicates @ standard error. Different letters indicate statistically significant differences (*p* ≤ 0.05) among treatments.

The relative expression of two putative Si transporter genes (*LpLsi1* and *LpLsi2*) in roots was assessed in ryegrass subjected to different Al and Si supplementation. In plants grown without Al, the expression level of *LpLsi1* and *LpLsi2* was down-regulated by approximately 4.2- and 2.8-fold, respectively, in response to Si addition to the growth media (**Figures 1A,B**). A similar expression pattern was observed when Al was applied alone, with the expression levels of *LpLsi1* and *LpLsi2* being reduced by approximately 7.1- and 2.9-fold, respectively (**Figures 1A,B**). However, when Al was added in combination with Si, the expression level of these Si transporters was significantly enhanced (**Figures 1A,B**). The highest Si dose applied to Al-treated plants increased the expression level of *LpLsi1* by approximately 5.4-fold (**Figure 1A**), whereas that of *LpLsi2* was up-regulated by at least 2.5-fold irrespective of Si dosage (**Figure 1B**).

### Lipid Peroxidation

**FIGURE 2:**
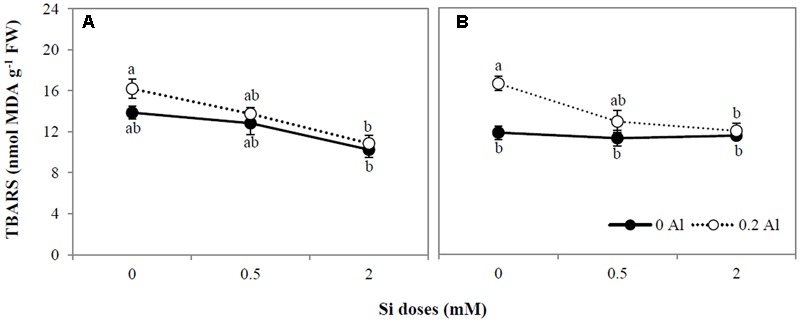
**Lipid peroxidation in shoot (A)** and root **(B)** of ryegrass hydroponically cultivated under Al and Si treatments. Data are means of three replicates ± standard error. Different letters indicate statistically significant differences (*p* ≤ 0.05) among treatments.

The addition of 0.2 mM Al increased root lipid peroxidation by approximately 29% (**Figure 2B**); however, no differences in oxidative damage were observed in shoots as a consequence of Al supply (**Figure 2A**). Likewise, no significant changes in TBARS accumulation were observed among plants grown with only Si (**Figures 2A,B**). However, Si at the highest concentration supplied diminished lipid peroxidation in Al-treated plants by approximately 32.6 and 27.7% in shoots and roots, respectively (**Figures 2A,B**). Consequently, lipid peroxidation was negatively correlated with Si concentration in shoots (*r* = -0.947, *p* ≤ 0.01) and roots (*r* = -0.734, *p* ≤ 0.05), as shown in **Table 3**.

### Plant Antioxidant Responses

**FIGURE 3:**
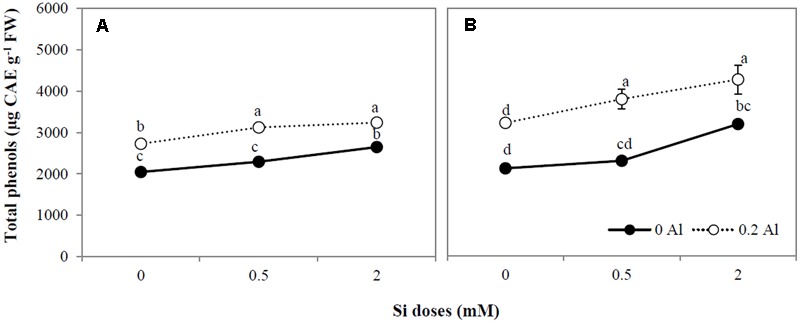
**Total phenol concentration in shoot (A)** and root **(B)** of ryegrass hydroponically cultivated under Al and Si treatments. Data are means of three replicates ± standard error. Different letters indicate statistically significant differences (*p* ≤ 0.05) among treatments.

Plants treated with Al showed an evident increment in total phenols (**Figures 3A,B**). A significant increase in total phenol concentration was also observed in the shoots and roots of ryegrass treated with the highest Si dose, with a further increase being observed in plants treated with both Al and Si (**Figures 3A,B**).

**FIGURE 4:**
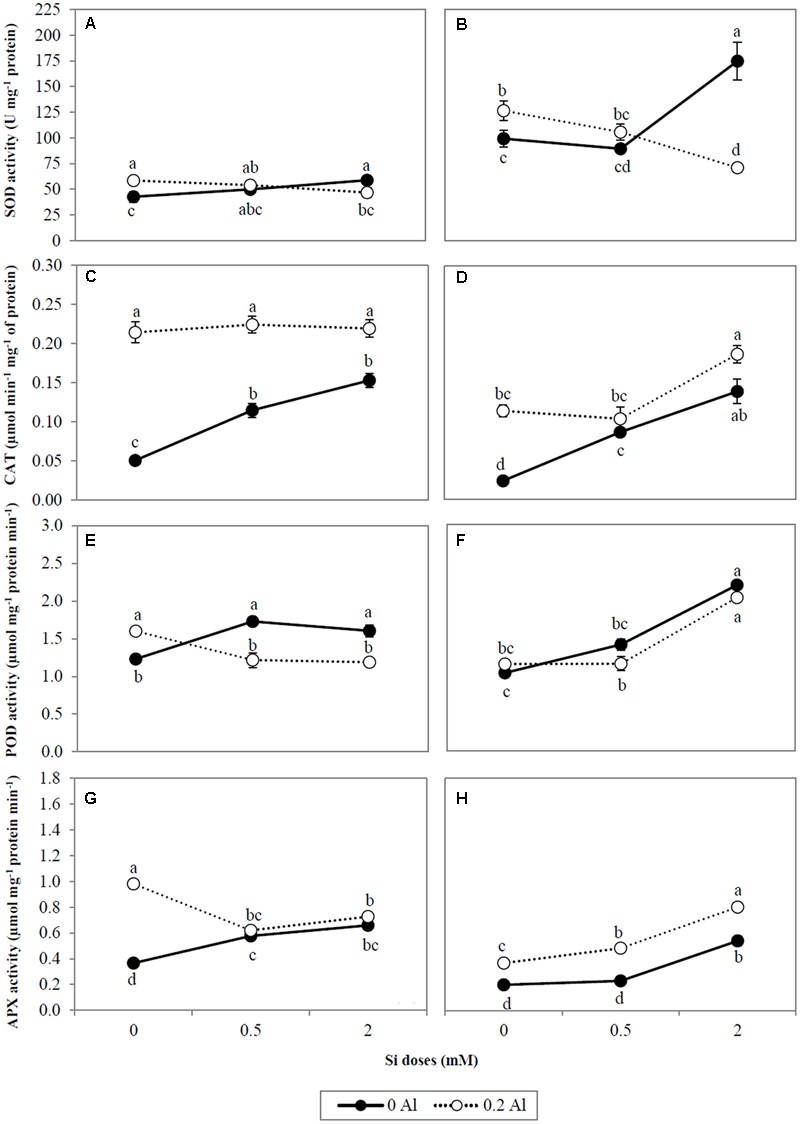
**The activity of antioxidant enzyme SOD (A,B)**, CAT **(C,D)**, POD **(E,F)**, and APX **(G,H)** in shoots and roots of ryegrass hydroponically cultivated under Al and Si treatments. Data are means of three replicates ± standard error. Different letters indicate statistically significant differences (*p* ≤ 0.05) among treatments.

In order to investigate the effect of Si on the ROS scavenging enzyme system under Al stress conditions, the activities of SOD, CAT, POD, and APX enzymes were evaluated (**Figures 4A–H**). Aluminum supplied alone significantly increased SOD activity by approximately 37.2% in shoots and 27.5% in roots (**Figures 4A,B**). Likewise, the highest Si dose activated SOD enzyme in non-Al-treated plants (**Figures 4A,B**). However, when Al and Si were simultaneously applied, SOD activity was significantly reduced by 20.08 and 43.8% in shoots and roots, respectively (**Figures 4A,B**).

The application of Al alone increased CAT activity in shoots and roots by at least 4.2- and 4.7-fold, respectively (**Figures 4C,D**). In plants grown in the absence of Al, Si enhanced CAT activity by approximately 3.0-fold (shoots) and 5.8-fold (roots) (**Figures 4C,D**). Plants supplied with Al + Si did not show significant differences in CAT activity compared with those supplied with Al alone, the exception being in the roots of plants supplied with the highest Si dose, which exhibited an approximate 60% increase (**Figures 4C,D**).

Shoot POD activity increased by approximately 30% in Al- treated plants compared with non-treated plants, although no significant changes were observed in roots (**Figures 4E,F**). The addition of Si augmented POD activity in plants grown without Al (**Figures 4E,F**). This effect was most evident in roots, in which the activity of this enzyme was increased by 2.1-fold at the highest Si supply (**Figure 4F**). Likewise, root POD was activated by approximately 1.7-fold under combined Al and Si treatments (**Figure 4F**), whereas in shoots the enzyme activity was diminished (**Figure 4E**).

Addition of Al to the growth media considerably increased APX activity by approximately 2.7-fold and 1.8-fold in shoots and roots, respectively (**Figures 4G,H**). Similarly, Si application elevated APX activity in ryegrass (**Figures 4G,H**), and this effect was enhanced by 2.2-fold in the roots of plants receiving the combined Al-Si treatments (**Figure 4H**). Conversely, Si supply decreased shoot APX activity by approximately 25.9% in Al-treated plants (**Figure 4G**).

The changes in antioxidant responses of Al-stressed plants as a consequence of Si uptake were additionally examined by means of Pearson correlation as shown in the **Table 3**. Briefly, we found a negative correlation between Si concentration and SOD activity in shoots (*r* = -0.827, *p* ≤ 0.01) and roots (*r* = -0.961, *p* ≤ 0.01). Conversely, for roots, we observed positive relationships between Si concentration and either total phenols (*r* = 0.741, *p* ≤ 0.05) or the antioxidant enzymes of the second line of defense (CAT, *r* = 0.838, *p* ≤ 0.01; POD, *r* = 0.925, *p* ≤ 0.01; APX, *r* = 0.980, *p* ≤ 0.01).

### Analysis of SOD Isoform Gene Expression in Response to Al and Si Treatments

**FIGURE 5:**
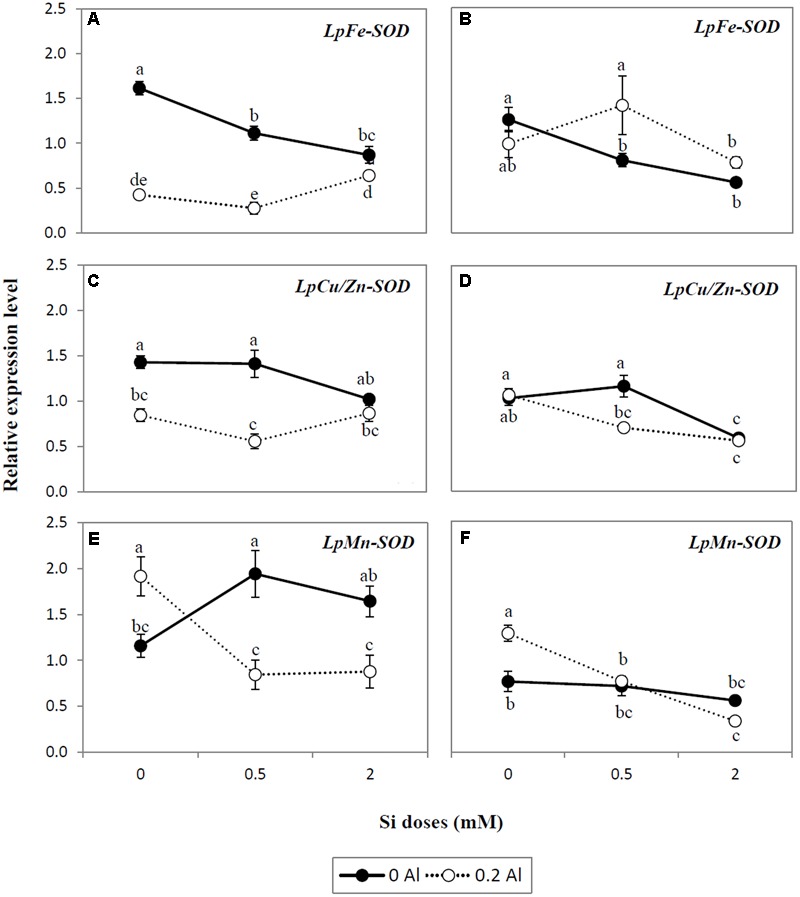
**Expression analysis of SOD isoform genes *LpFe-SOD* (A,B)**, *Cu/Zn-SOD* **(C,D)**, and *Mn-SOD* **(E,F)** determined by qRT-PCR in shoots and roots of ryegrass hydroponically cultivated under Al and Si treatments. The expression levels were normalized in relation to *Actin* or *eEF1A(m)* gene expression. Data are means of three replicates ± standard error. Different letters indicate statistically significant differences (*p* ≤ 0.05) among treatments.

Genes of SOD isoforms (Fe-SOD, Cu/Zn-SOD, and Mn-SOD) were differentially expressed as a consequence of Si and Al supply (**Figures 5A–F**). Aluminum supplied alone reduced the gene expression of Fe-SOD and Cu/Zn-SOD in shoots (**Figures 5A,C**), whereas no changes in the expression pattern of these genes was detected in the roots (**Figures 5B,D**). In addition, expression of the Mn-SOD gene was up-regulated by approximately 1.7-fold in shoots and roots exposed to Al (**Figures 5E,F**). Increasing Si doses lowered the gene expression of Fe-SOD by up to 1.9-fold in the shoots and 2.2-fold in the roots of plants cultivated without Al (**Figures 5A,B**), whereas the transcript levels of Mn-SOD were enhanced in shoots by approximately 1.7-fold by Si addition (**Figure 5E**). In contrast, in plants receiving Si alone, there was no significant changes in the expression level of either shoot Cu/Zn-SOD or root Mn-SOD genes (**Figures 5C,F**). However, in roots, Cu/Zn-SOD was down-regulated by at least 1.8-fold as a consequence of Si supply (**Figure 5D**). In plants simultaneously exposed to Al and Si, the addition of Si did not induce significant changes in the expression level of Fe-SOD in shoots and roots (**Figures 5A,B**). Although a similar expression pattern of Cu/Zn-SOD was observed in the shoots of Al-treated plants under the different Si treatments (**Figure 5C**), the gene expression of this enzyme was down-regulated by up to 1.9-fold in roots (**Figure 5D**). Likewise, Si application to Al-treated plants significantly reduced the transcript level of Mn-SOD by at least 2.2- and 3.8-fold in shoots and roots, respectively (**Figures 5E,F**).

### Hydrogen Peroxide Production in Shoot Protoplasts Exposed to Al and Si

**FIGURE 6:**
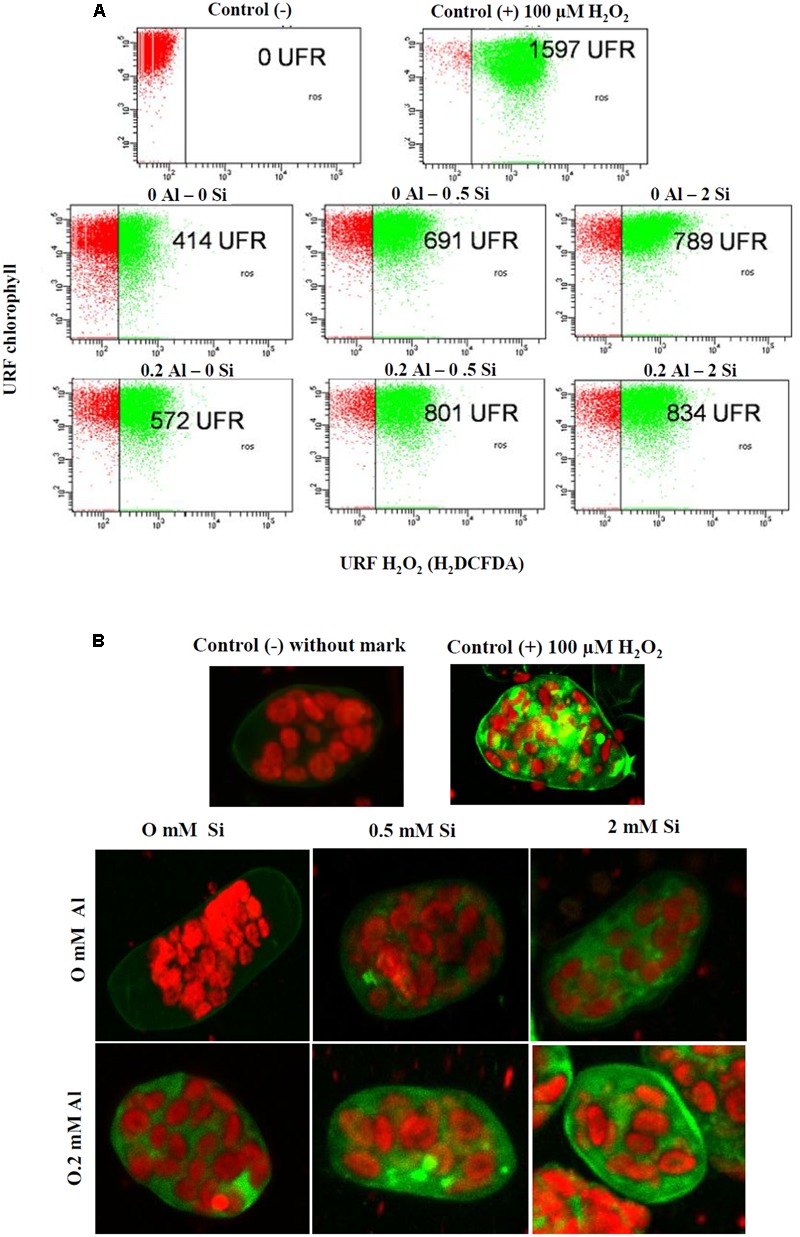
**Hydrogen peroxide (H_2_O_2_) production in shoot protoplasts of ryegrass hydroponically cultivated under Al and Si treatments. (A)** Dot plot representation of flow cytometry data. For the positive control, 100 μM H_2_O_2_ was used. **(B)** Confocal projection images showing the increasing concentration of H_2_O_2_. Hydrogen peroxide fluorescence were collected by excitation/emission wave lengths 488 nm/530 nm by Confocal Laser Scanning Microscope.

Aluminum treatment augmented H_2_O_2_ generation by approximately 38% in shoot protoplasts (**Figure 6A**). A progressive increase in H_2_O_2_ production was also observed when Si was added alone, and the accumulation of H_2_O_2_ was enhanced to an even greater extent in plants simultaneously supplied with Si and Al (**Figure 6A**). This pattern was consistent with the observations made by CLSM analysis (**Figure 6B**), which revealed a progressive increase in the fluorescence of an H_2_DCFDA probe generated by Si and Al application.

## Discussion

Although several previous studies have reported that Si provide beneficial effects on plants subjected to Al stress, the mechanisms underlying these responses have remained poorly understood. Moreover, only a few studies have examined the effect of Si-mediated amelioration of Al toxicity in terms of the regulation of Al and Si uptake systems (e.g., Britez et al., 2002; Wang et al., 2004; Dorneles et al., 2016) and plant antioxidant performance (e.g., Shahnaz et al., 2011; Shen et al., 2014). Likewise, to date, the effect of Si on Al stress in ryegrass, a forage species belonging to Si-accumulator plants (Jarvis, 1987; Nanayakkara et al., 2008), has yet to be addressed.

The high level of toxic Al in acid soils is an important limiting factor for plant production (Mora et al., 2006). In our study, the exposure of plants to 0.2 mM Al significantly increased Al accumulation, mainly in the roots (**Table 2**), with a consequent reduction of approximately 28.5% in root dry matter production (**Table 2**). These results are consistent with our previous findings for ryegrass (Cartes et al., 2010), since it is well known that Al toxicity involves the rapid inhibition of root growth (e.g., Matsumoto, 2000; Kochian et al., 2005; Horst et al., 2010; Singh et al., 2017). The role played by Si in promoting plant growth under Al toxicity has been widely accepted (e.g., Hara et al., 1999; Singh et al., 2011; Shen et al., 2014; Tripathi et al., 2016). Correspondingly, Si application to Al-treated plants significantly reduced the Al concentration in ryegrass (**Table 2**) and improved root dry weight by at least 51% (**Table 2**). A slight reduction in Si concentration in plant tissues was also found when plants were simultaneously supplied with Al and Si (**Table 2**). Moreover, our results revealed a negative correlation between Si and Al uptake in plants treated with Al and Si, whereas Si concentration and dry matter production were positively related (**Table 3**). The reduction in Al and Si uptake might be attributed to the formation of biologically inactive aluminosilcate (Al-Si) complexes in the growth media, thus lowering Al availability (Barcelo et al., 1993; Baylis et al., 1994; Ma et al., 1997; Cocker et al., 1998a), with the consequent enhancement of root growth. Nevertheless, the formation of Al-Si inside plant tissues could also be involved in the growth-promoting effect of Si under Al stress (Hodson and Sangster, 1993; Cocker et al., 1998b; Wang et al., 2004). Indeed, it has been demonstrated that Al toxicity may be decreased by co-deposition of Al and Si in the root epidermal walls of sorghum (Hodson and Sangster, 1993). Similarly, Cocker et al. (1998b) and Wang et al. (2004) have also suggested that formation of Al-Si complexes in the root apoplast of wheat and maize is a possible mechanism for Al detoxification in plants.

Although all plants contain Si in their tissues, the concentration of this element varies greatly among species, in a range from 0.1 to 10% on a dry weight basis (Epstein, 1999; Ma and Takahashi, 2002), which is indicative of the fact that the benefits of Si to plants grown under stress can also be highly variable. Recent studies have shown that Si accumulation in plants is controlled by influx and efflux Si transporters that could be involved in the differential Si-induced responses to cope with different plant stress (e.g., Ma et al., 2006, 2007; Yamaji et al., 2008, 2012; Chiba et al., 2009; Mitani et al., 2009a,b, 2011a,b; Yamaji and Ma, 2009; Grégoire et al., 2012; Montpetit et al., 2012; Deshmukh et al., 2013; Ma and Yamaji, 2015). To further investigate the effect of Si uptake on ryegrass subjected to Al stress, we assessed the gene expression of two Si transporters (*Lsi1* and *Lsi2*) in plants with different Al and Si supply (**Figures 1A,B**). Lsi1 is a channel-type transporter belonging to aquaporin Nodulin26-like intrinsic protein (NIP) III subfamily (Ma et al., 2006), whereas Lsi2 is an Si efflux transporter belonging to the family of putative anion transporters (Ma et al., 2007). Efficient coupling of Lsi1 with Lsi2 controls the uptake of Si in species such as rice, barley, and maize (Ma et al., 2006, 2007; Chiba et al., 2009; Mitani et al., 2009a,b). Our study showed that in plants cultivated without Al, the mRNA expression levels of both *LpLsi1* and *LpLsi2* were down-regulated in plants supplied with Si (**Figures 1A,B**). Some studies have shown that the accumulation of *Lsi1* mRNA in maize (*ZmLsi1)*, barley (*HvLsi1)*, and wheat (*TaLsi1*) is not affected by the addition of Si (Chiba et al., 2009; Mitani et al., 2009a; Montpetit et al., 2012). Nevertheless, Ma et al. (2006, 2007) found that the gene expression of both *OsLsi1* and *OsLsi2* was decreased by approximately 25% in rice, as a consequence of continuous Si application. A similar expression pattern has been detected for *Lsi1* in maize (*ZmLsi1*) (Bokor et al., 2014) as well as for *Lsi2* in barley (*HvLsi2*) (Mitani et al., 2009b) and maize (Bokor et al., 2014). Moreover, a recent study has stated that the Si-induced down-regulation of Si transporter genes is controlled by Si accumulation in the shoots of rice (Mitani et al., 2016).

At present, there is little information on the effect of any plant stress on the transcriptional regulation of Si transporters genes. Bokor et al. (2014) observed that Si supply down-regulated the expression of *ZmLsi1* and *ZmLsi2* in the roots of maize subjected to excess zinc (Zn). By contrast, it has been reported that Si increased the expression level of *OsLsi1* and *OsLsi2* under conditions of cadmium (Cd) and copper (Cu) toxicity in rice plants (Kim et al., 2014). Likewise, Vulavala et al. (2016) found that a putative Si transporter in potato (*StLsi1*) was up-regulated in response to Si and drought stress. Interestingly, we found that the transcript levels of both *LpLsi1* and *LpLsi2* were significantly down-regulated by Al supply, but up-regulated by 5.4-fold (*LpLsi1*) and 2.5-fold (*LpLsi2*) when Al was added in combination with Si (**Figures 1A,B**). Compared with plants cultivated with Si alone, the reduction in Si concentration in plants simultaneously supplied with Al and Si (**Table 2**), could be responsible for the up-regulation of *LpLsi1* and *LpLsi2* (**Figures 1A,B**). This behavior might indicate an increased requirement for Si in ryegrass in order to cope with Al-induced toxicity. Further studies are needed to confirm this assumption.

As a possible alternative mechanism of Si-mediated Al detoxification in plants, enhancement of the antioxidant defense system has also been proposed (Shahnaz et al., 2011; Shen et al., 2014; Liang et al., 2015; Tripathi et al., 2016). As stated above, Al toxicity can lead to the generation of reactive oxygen species (ROS), such as superoxide radicals (O_2_^•-^), hydroxyl radicals (^•^OH), and hydrogen peroxide (H_2_O_2_) molecules, which cause oxidative damage to plant cells (e.g., Yamamoto et al., 2001, 2002, 2003; Kochian et al., 2005; Singh et al., 2017). In agreement with previous reports (Cartes et al., 2010, 2012), our results show that 0.2 mM Al increased lipid peroxidation in ryegrass (**Figures 2A,B**), confirming that oxidative stress occurs under Al supply. Nevertheless, 2 mM Si significantly diminished Al-induced lipid peroxidation by approximately 32 and 28% in shoots and roots, respectively (**Figures 2A,B**). Moreover, a negative correlation between Si concentration and lipid peroxidation was detected in Al-treated plants (**Table 3**). Consistent with our findings, Shen et al. (2014) observed a noticeable decrease in lipid peroxidation attributable to Si in peanut grown under Al excess. Similarly, there is increasing evidence showing that oxidative damage to biological membranes decreases as a consequence of Si application to plants subjected to different environmental stresses (e.g., Liang et al., 2003; Zhu et al., 2004; Shi et al., 2005; Gunes et al., 2007, 2008; Li et al., 2012; Khoshgoftarmanesh et al., 2014; Kim et al., 2014; Habibi, 2015; Zia-ur-Rehman et al., 2016).

Whereas Al toxicity enhanced plant phenols concentration (**Figures 3A,B**) and augmented the activities of antioxidant enzymes (**Figures 4A–H**), Si application induced differential responses in the antioxidant system of Al-stressed plants (**Figures 3A,B, 4A–H**). It has been suggested that Si may enhance Al tolerance by increasing the production of phenolic compounds with Al-chelating ability (Kidd et al., 2001; Shahnaz et al., 2011). Furthermore, it has been reported that Si uptake by plants subjected to certain stresses can lead to increased production of phenolics with antioxidant and/or structural function (Fleck et al., 2010, 2015; Song et al., 2016). Likewise, enzymes and genes involved in the biosynthesis of either soluble phenolics (e.g., flavonoids) or structural polyphenols (e.g., lignin) have also been shown to be induced by Si (Liang et al., 2007; Shetty et al., 2011; Zhang et al., 2013; Song et al., 2016). Here, we found that Si addition (mainly at the highest dose) increased the total phenol concentration in plants treated with Al and Si (**Figures 3A,B**), and that there was a negative relationship between phenols concentration and lipid peroxidation (**Table 3**). Thus, the enhanced phenols accumulation triggered by Si may have contributed to the amelioration of Al-induced oxidative stress in ryegrass.

Differential changes in the activity of antioxidant enzymes, as a consequence of Al and Si treatments, were also observed. SOD constitutes the first line of defense in the enzymatic antioxidant responses by catalyzing the dismutation of O_2_^•-^ to H_2_O_2_ and O_2_ (Takahashi and Asada, 1983; Alscher et al., 2002). Our results indicate that the highest Si dose decreased SOD activity in plants subjected to Al stress (**Figures 4A,B**), as supported by the negative correlation between SOD activity and Si concentration (**Table 3**). Likewise, differential gene expression of SOD isoforms occurred in plants exposed to Al and Si (**Figures 5A–F**). The major differences were detected in the roots at the highest Si level, which induced a significant decrease in expression of the *LpCu/Zn-SOD* and *LpMn-SOD* genes in plants grown under Al toxicity (**Figures 5D,F**). A similar expression pattern was observed for the *LpMn-SOD* gene in shoots, which was down-regulated by at least 2.2-fold in combined Al-Si treatments (**Figure 5E**). The decrease in either SOD activity (**Figures 4A,B**) or the gene expression pattern of SOD isoforms (**Figures 5A,D,F**) coincided with a significant reduction in lipid peroxidation at the highest Si dose (**Figures 2A,B**), denoting that 2 mM Si can diminish the requirement for SOD enzyme in Al-treated plants.

It is noteworthy that the antioxidant enzymes responsible for H_2_O_2_ scavenging (CAT, POD, and APX) were activated by Si in the roots of Al-stressed plants (**Figures 4D,F,H**). Moreover, a direct correlation between Si concentration and the activities of CAT, POD, and APX was found in the roots of plants treated with Al and Si (**Table 3**). The activation of these enzymes was accompanied by a noticeable decrease in lipid peroxidation (**Figures 2A,B**), with a consequent reduction in the oxidative damage of biological membranes induced by Al.

We also detected an apparent increase in intracellular H_2_O_2_ production in shoot protoplasts of plants simultaneously supplied with Al and Si (**Figures 6A,B**). It is remarkable that there is so little information available regarding the role of Si in H_2_O_2_ generation under either biotic or abiotic stress conditions. In this context, the only study that has examined the relationship between Si and H_2_O_2_ production in plants subjected to Al toxicity (Lima et al., 2016) showed an opposite trend when compared with our results. Nevertheless, under freezing stress, Habibi (2015) detected an increase in H_2_O_2_ levels induced by Si in pistachio plants, which is consistent with the findings of the present study. This significant increase in H_2_O_2_ production might be related to the reduction in POD activity observed in the shoots of plants simultaneously treated with Al and Si (**Figure 4E**). Indeed, H_2_O_2_ plays a dual role in vascular plants by either inducing oxidative damage or acting as signaling molecule in several physiological processes, including senescence (Peng et al., 2005), photorespiration and photosynthesis (Noctor and Foyer, 1998), and growth and development (Foreman et al., 2003). H_2_O_2_ also functions as a second messenger that modulates the expression of antioxidant enzymes and stress responses (Apel and Hirt, 2004). Accordingly, further work should focus on the mechanisms underlying the Si modulation of H_2_O_2_ production under Al stress.

Finally, taken together, our findings provide the first biochemical and molecular evidence that Si counteracts the negative effects of Al by modulating Al and Si uptake as well as enzymatic and non-enzymatic antioxidant responses in ryegrass plants.

## Author Contributions

SP and PC conceived the idea and wrote the manuscript. SP performed all the experiments and PC supervised the research. AG-M and HJ contributed to evaluation and discussion regarding aspects of the study related to gene expression analyses. KG assisted with management and analysis of the flow cytometry and laser scanning CLSM data. MM contributed to discussion on aspects associated with the influence of Si on plants subjected to Al toxicity. All authors contributed to the discussion and approved the final manuscript.

## Conflict of Interest Statement

The authors declare that the research was conducted in the absence of any commercial or financial relationships that could be construed as a potential conflict of interest.
